# Weak temperature dependence of *P*^+^*H*_A_^−^ recombination in mutant *Rhodobacter sphaeroides* reaction centers

**DOI:** 10.1007/s11120-016-0239-9

**Published:** 2016-03-04

**Authors:** Krzysztof Gibasiewicz, Rafał Białek, Maria Pajzderska, Jerzy Karolczak, Gotard Burdziński, Michael R. Jones, Klaus Brettel

**Affiliations:** Department of Physics, Adam Mickiewicz University, ul. Umultowska 85, 61-614 Poznań, Poland; Center for Ultrafast Laser Spectroscopy, A. Mickiewicz University, ul. Umultowska 85, 61-614 Poznań, Poland; School of Biochemistry, Medical Sciences Building, University of Bristol, University Walk, Bristol BS8 1TD UK; Laboratoire Mécanismes Fondamentaux de la Bioénergétique, UMR 8221, CEA - iBiTec-S, CNRS, Université Paris Sud, 91191 Gif-Sur-Yvette, France

**Keywords:** *Rhodobacter sphaeroides*, Reaction centers, Charge recombination, Electron transfer, Transient absorption, Protein dynamics

## Abstract

In contrast with findings on the wild-type *Rhodobacter sphaeroides* reaction center, biexponential *P*^+^*H*_A_^−^ → *PH*_A_ charge recombination is shown to be weakly dependent on temperature between 78 and 298 K in three variants with single amino acids exchanged in the vicinity of primary electron acceptors. These mutated reaction centers have diverse overall kinetics of charge recombination, spanning an average lifetime from ~2 to ~20 ns. Despite these differences a protein relaxation model applied previously to wild-type reaction centers was successfully used to relate the observed kinetics to the temporal evolution of the free energy level of the state *P*^*+*^*H*_*A*_^*−*^ relative to *P*^+^*B*_A_^−^. We conclude that the observed variety in the kinetics of charge recombination, together with their weak temperature dependence, is caused by a combination of factors that are each affected to a different extent by the point mutations in a particular mutant complex. These are as follows: (1) the initial free energy gap between the states *P*^+^*B*_A_^−^ and *P*^+^*H*_A_^−^, (2) the intrinsic rate of *P*^+^*B*_A_^−^ → *PB*_A_ charge recombination, and (3) the rate of protein relaxation in response to the appearance of the charge separated states. In the case of a mutant which displays rapid *P*^+^*H*_A_^−^ recombination (ELL), most of this recombination occurs in an unrelaxed protein in which *P*^+^*B*_A_^−^ and *P*^+^*H*_A_^−^ are almost isoenergetic. In contrast, in a mutant in which *P*^+^*H*_A_^−^ recombination is relatively slow (GML), most of the recombination occurs in a relaxed protein in which *P*^+^*H*_A_^−^ is much lower in energy than *P*^+^*H*_A_^−^. The weak temperature dependence in the ELL reaction center and a YLH mutant was modeled in two ways: (1) by assuming that the initial *P*^+^*B*_A_^−^ and *P*^+^*H*_A_^−^ states in an unrelaxed protein are isoenergetic, whereas the final free energy gap between these states following the protein relaxation is large (~250 meV or more), independent of temperature and (2) by assuming that the initial and final free energy gaps between *P*^+^*B*_A_^−^ and *P*^+^*H*_A_^−^ are moderate and temperature dependent. In the case of the GML mutant, it was concluded that the free energy gap between *P*^+^*B*_A_^−^ and *P*^+^*H*_A_^−^ is large at all times.

**Electronic supplementary material:**

The online version of this article (doi:10.1007/s11120-016-0239-9) contains supplementary material, which is available to authorized users.

## Introduction

The contributions of protein dynamics to electron transfer occurring inside proteins are poorly understood. Photosynthetic reaction centers (RCs) are convenient model systems that allow systematic studies of this issue; they can be synchronously excited by ultrashort laser pulses, and the electron transfer may be observed by optical time-resolved techniques on time scales comparable with the duration of the excitation pulse and longer. Experimental data relating to the influence of protein dynamics on light-induced electron transfer in RCs have been interpreted either in terms of an active contribution of the protein, the spontaneous diffusion of which in the conformational space is a factor that largely determines the rate of electron transfer (Wang et al. 2007, 2009, 2012; Torchała and Kurzyński 2008; Pieper and Renger 2009; Kurzyński and Chełminiak 2013) or in terms of passive conformational reorganization of the protein in response to the appearance of a strong electrical field caused by the light-induced charge separated states (Woodbury and Parson 1984; Peloquin et al. 1994; Gibasiewicz et al. 2013a).

**Fig. 1:**
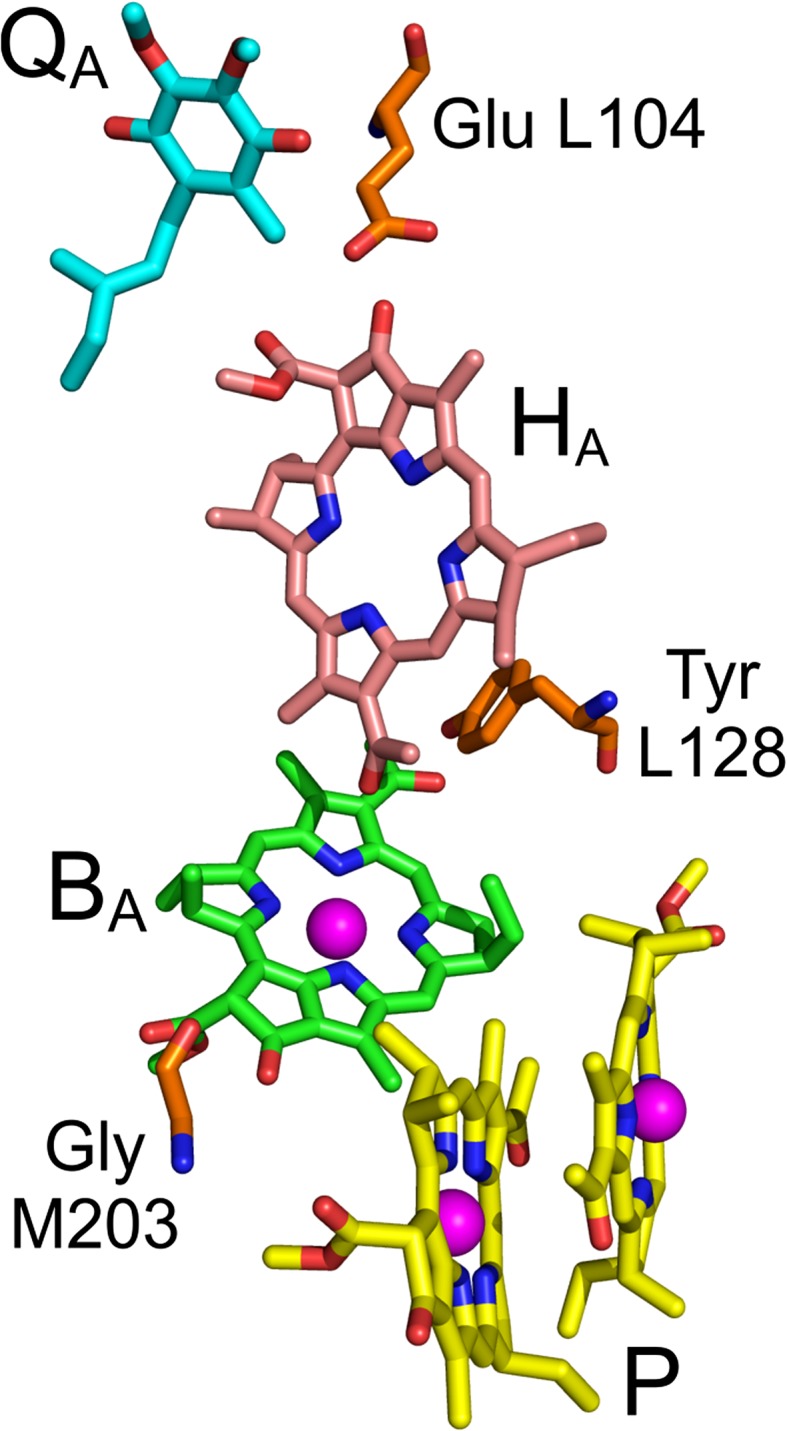
Electron transfer cofactors of *Rba. sphaeroides* RC. The primary electron donor P BChls are shown with yellow carbons, *B*_A_ acceptor BChl–with green carbons, *H*_A_ acceptor BPhe–with pink carbons and *Q*_A_ acceptor ubiquinone–with cyan carbons. Amino acids replaced by mutation are shown with orange carbons. For other atoms, *red* = oxygen, *blue* = nitrogen, and *magenta* sphere = magnesium

Among the photosynthetic RCs, one of the most widely studied is that from the purple bacterium *Rhodobacter* (*Rba.*) *sphaeroides* (Woodbury and Allen 1995; Zinth and Wachtveitl 2005; Hunter et al. 2009; Jones 2009). Light-induced charge separation inside this protein occurs along a chain of electron transfer cofactors that engage in non-covalent interactions with the neighboring amino acids. The electron transfer starts at the primary electron donor, a dimer of bacteriochlorophyll *a* (BChl *a*) molecules, labeled traditionally as P870 or P (Fig. 1). Within ~3–7 ps, the electron is transferred from the first excited singlet state of *P* (P*) to *H*_A_, a bacteriopheophytin (Fig. 1), forming the first spectroscopically easily observed charge separated state *P*^+^*H*_A_^−^. Then, the electron is transferred within ~200–250 ps from *H*_A_^−^ to the secondary electron acceptor, a quinone named *Q*_A_ (Fig. 1), forming the charge separated state, *P*^+^*Q*_A_^−^. From *Q*_A_, the electron is transferred on microsecond time scale to a dissociable quinone, *Q*_B_. The forward electron transfer from *H*_A_^−^ to *Q*_A_ can be artificially blocked by chemical or genetic removal of *Q*_A_ (Shuvalov and Parson 1981; Schenck et al. 1982; Chidsey et al. 1984; Ogrodnik et al. 1988; Tang et al. 1999; Ridge et al. 1999; McAuley et al. 2000), or by pre-reduction of *Q*_A_ to *Q*_A_^−^ using a strong reductant, or through a combination of weak background illumination (forming the long-lived state *P*^+^*Q*_A_^−^) and a relatively weak reductant (reducing *P*^+^ to leave *PQ*_A_^−^) (Shuvalov and Parson 1981; Schenck et al. 1982; Gibasiewicz and Pajzderska 2008; Gibasiewicz et al. 2009). It cannot be excluded that in nature, under certain conditions, the state *PQ*_A_^−^ may also be formed. When the electron transfer from *H*_A_^−^ to *Q*_A_ is blocked, a charge recombination occurs in which the electron is transferred back to *P*^+^ (Volk et al. 1995), and this charge recombination may be measured directly.

**Fig. 2:**
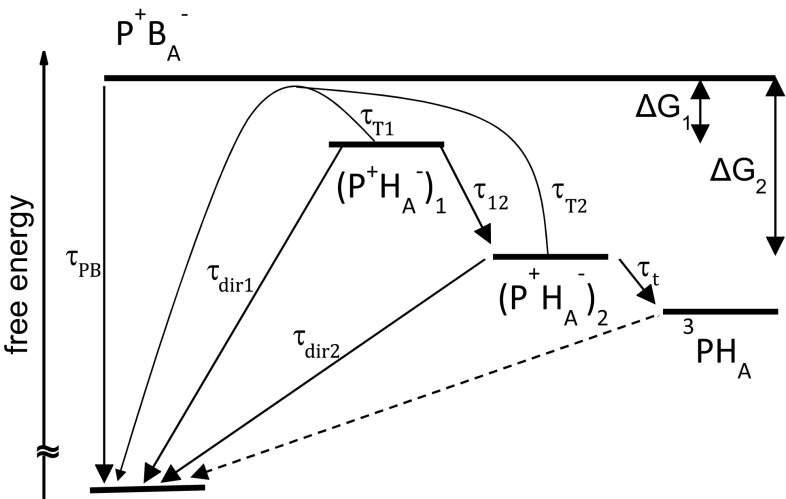
Model scheme of *P* ^+^ *H*_A_^−^ recombination in the case of one-step protein relaxation. Lifetimes *τ*_T1_ and *τ*_T2_ are temperature dependent and depict thermally activated charge recombination with real formation of the intermediate state *P* ^+^ *B*_A_^−^, whereas lifetimes *τ*_dir1_ and *τ*_dir2_ are temperature independent and depict direct *P* ^+^ *H*_A_^−^ recombination with virtual involvement of *B*_A_. The free energy gap between *P* ^+^ *H*_A_^−^ and *P* ^+^ *B*_A_^−^ increases in time from Δ*G*_1_ to Δ*G*_2_, with a lifetime *τ*_12_, due to protein reorganization (solvation) dynamics in response to the appearance of the charge separated states (Gibasiewicz et al. 2013a). Thus, (*P* ^+^ *H*_A_^−^)_1_ is the initial or unrelaxed form and (*P* ^+^ *H*_A_^−^)_2_ is the final or relaxed form of the charge separated state, and both are assumed to be in equilibrium with *P* ^+^ *B*_A_^−^. Relaxation of *P* ^+^ *H*_A_^−^ underlies the non-mono-exponential character of *P* ^+^ *H*_A_^−^ recombination. The values of *τ*_T1_ and *τ*_T2_ depend on Δ*G*_1_, Δ*G*_2_, and *τ*_PB_: *τ*_Ti_ = *τ*_PB_[1 + exp(Δ*G*_*i*_/*kT*)], *i* = 1, 2 (compare Appendix in the Supporting Information). Formation of the triplet state ^3^ *PH*_A_ competes with recombination to the singlet states of P. Recombination to the singlet excited state P* is not shown, since it was assumed that depopulation of the state *P* ^*+*^ *H*_A_^−^ via fluorescence channel is much less efficient than those depicted in the scheme

The *B*_A_ BChl located between *P* and *H*_A_ (Fig. 1) acts as either a real or virtual intermediate electron carrier for both forward electron transfer from P* to *H*_A_ (Kirmaier and Holten 1988, 1991; Lockhart et al. 1990; Rodriguez et al. 1991; Chan et al. 1991; Arlt et al. 1993) and backward transfer from *H*_A_^−^ to *P*^+^ (Shuvalov and Parson 1981; Shkuropatov and Shuvalov 1993; Schmidt et al. 1994; Kirmaier et al. 1995; Gibasiewicz and Pajzderska 2008; Wang et al. 2012). Possible pathways for *P*^+^*H*_A_^−^ recombination are summarized in Fig. 2. Involvement of *B*_A_ as a real carrier in charge recombination means that a discrete *P*^+^*B*_A_^−^ state is formed before the electron arrives at *P*^+^. Experimental data have shown that the energy level of *P*^+^*B*_A_^−^ is higher than that of *P*^+^*H*_A_^−^ (Shuvalov and Parson 1981; Arlt et al. 1993; Heller et al. 1996) and therefore the efficiency of this charge recombination pathway is temperature dependent (Gibasiewicz et al. 2013a). In contrast, the virtual involvement of B_A_ in charge recombination through superexchange provides an alternative pathway for this reaction that is temperature independent. Irrespective of mechanism, the charge recombination reaction leads with the highest probability to energy dissipation and immediate recovery of the singlet ground state of P. However, in *Q*_A_-reduced wild-type (WT) RCs at room temperature there is a ~15 % probability that the spin of one of the radical pair electrons will change in the state *P*^+^*H*_A_^−^ leading to a triplet configuration of the charge separated state, ^3^(*P*^+^*H*_A_^−^), followed by charge recombination yielding the triplet state of the primary donor ^3^*P* (Volk et al. 1995). Triplet states decay on microsecond time scale (Shuvalov and Parson 1981; Woodbury et al. 1986; Volk et al. 1995; Gibasiewicz et al. 2011). Finally, there is a small probability that the electron on *H*_A_^−^ will come back to the excited orbital of the primary donor regenerating the singlet excited state P* (Woodbury and Parson 1984; Woodbury et al. 1986; Ogrodnik et al. 1994; Hartwich et al. 1998).

It has been hypothesized that *P*^+^*H*_A_^−^ recombination is strongly influenced by protein dynamics (Gibasiewicz et al. 2011, 2013a; Wang et al. 2012), the main experimental evidence being the multiexponential character of this reaction. In the model shown in Fig. 2, the transition indicated by the arrow and label *τ*_12_ depicts a downshift of the energy level of *P*^+^*H*_A_^−^ relative to *P*^+^*B*_A_^−^ ascribed previously to protein conformational adaptation to the appearance of the new charges on P^+^ and H_A_^−^ (Gibasiewicz et al. 2013a). In membrane-bound WT *Rba. sphaeroides* RCs, the overall characteristic lifetime of this process was estimated to be *τ*_12_ = 3.7 ± 0.4 ns with no evident temperature dependence between 78 K and room temperature (RT) (Gibasiewicz et al. 2013b). For isolated *Rba. sphaeroides* RCs, it was possible to resolve as many as three exponential components in transient absorption measurements due to a better signal-to-noise ratio. Those results were treated with a similar model to that one shown in Fig. 2, but with two consecutive steps of protein relaxation that lower the free energy of *P*^+^*H*_A_^−^ with lifetimes of *τ*_12_ = 0.6 ± 0.1 ns and *τ*_23_ = 10.5 ± 1.5 ns (not shown in Fig. 2). Again, no clear temperature dependence of these parameters was observed between 77 K and RT (Gibasiewicz et al. 2013a). The single value of *τ*_12_ = 3.7 ns obtained for membrane-bound RCs falls in between the *τ*_12_ = 0.6 ns and *τ*_23_ = 10.5 ns obtained for isolated RCs and could be regarded as an average value.

Population of the state *P*^+^*B*_A_^−^ in the WT RC is very low compared to that of *P*^+^*H*_A_^−^, and thus *B*_A_^−^ is very difficult to detect (Arlt et al. 1993; Woodbury and Allen 1995; Parson and Warshel 2009). However, manipulation of the free energy gap between *P*^+^*B*_A_^−^ and *P*^+^*H*_A_^−^ through genetic modification increased the amount of *P*^+^*B*_A_^−^ to detectable levels (Kirmaier et al. 1991, 1995; Shkuropatov and Shuvalov 1993; Laporte et al. 1995; Heller et al. 1995, 1996; Schmidt et al. 1994, 1995; Huber et al. 1995; Arlt et al. 1996; Kennis et al. 1997). Interestingly, a *P*^+^*B*_A_^−^ state in equilibrium with *P*^+^*H*_A_^−^ was reported also for WT RCs both on the time scale of primary charge separation (a few picoseconds) and *P*^+^*H*_A_^−^ → *PH*_A_ charge recombination (~1 ns) (Arlt et al. 1993; Gibasiewicz et al. 2009; Zhu et al. 2013). Using femtosecond transient absorption measurements in the visible region, it was shown that contribution of the absorption increased at ~544 nm (due to *P*^+^*H*_A_^−^ → *PH*_A_ charge recombination; at ~544 nm only the signal from *H*_A_^−^/*H*_A_ contributes) relative to that at ~600 nm (due to *P*^+^*B*_A_^−^ → *PB*_A_ charge recombination; at ~600 nm both signals from *P*/*P*^+^ and *B*_A_^−^/*B*_A_ contribute) was dependent on the presence/absence of the negative charge on *Q*_A_ and on the time delay between excitation and measurement (Gibasiewicz et al. 2009). At shorter times after excitation (below 1 ns) and/or with a negative charge on *Q*_A_, the charge recombination was more pronounced at ~600 nm, whereas at later times and/or without negative charge on *Q*_A_ (in practice—without *Q*_A_), the charge recombination was more pronounced at ~544 nm. It was therefore concluded that the bigger the charge recombination signal at ~600 nm relative to that at ~544 nm, the more the equilibrium between the states *P*^+^*H*_A_^−^ and *P*^+^*B*_A_^−^ shifts toward the latter and the smaller is the free energy gap between these states. Therefore, the relative amplitudes of the charge recombination signals at these two wavelengths may be treated as a fingerprint of this free energy gap (see also Heller et al. 1996). This finding will be particularly useful in interpretation of the femtosecond data for the mutants presented in the Results and Discussion.

Recently, it was proposed that charge separation may occur via two parallel pathways characterized by somewhat different electron transfer rates from P* to *B*_A_ and from *B*_A_^−^ to *H*_A_, possibly due to different conformational states of the protein (Zhu et al. 2013) in line with older work reporting on heterogeneity of the charge separation reaction (Kirmaier and Holten 1990). Although we cannot rule out the possibility that similar heterogeneity underlies multiexponential charge recombination, in this contribution we limit our modeling to homogenous situation. Obviously, postulated structural heterogeneity occurring on the time scale of charge separation does not need to be sustained to the nanosecond time scale on which charge recombination occurs. A longer discussion on the issue of heterogeneity of charge recombination can be found in a previous report (Gibasiewicz et al. 2013a).

In this contribution, we compared the temperature dependence of the kinetics of *P*^+^*H*_A_^−^ charge recombination in membrane-bound WT and three single point mutant RCs with identically blocked electron transfer from *H*_A_^−^ to *Q*_A_ achieved by pre-reduction of the latter using background illumination and a weak reductant, sodium ascorbate. It is proposed that these mutations, which are known to reorganize hydrogen bond interactions either with *H*_A_ or with *B*_A_ (Bylina et al. 1988; Potter et al. 2005; Gibasiewicz et al. 2011), modulate not only the free energy levels of the state *P*^+^*H*_A_^−^ relative to that of *P*^+^*B*_A_^−^, as was proposed previously, but also the responsive protein dynamics. Consequently, both of these factors, as well as the intrinsic *P*^+^*B*_A_^−^ → *PB*_A_ charge recombination rate constant, are responsible for the observed variety of charge recombination kinetics and the weak temperature dependence of *P*^+^*H*_A_^−^ charge recombination.

## Materials and methods

### Biological material

Strains of *Rba. sphaeroides* lacking both types of light-harvesting complex and containing either WT or mutated RCs were grown under dark/semiaerobic conditions as described previously (Jones et al. 1992). Cells were harvested and intracytoplasmic membranes isolated by breakage of cells in a French pressure cell, followed by sucrose gradient purification (Jones et al. 1994).

### Nanosecond transient absorption measurements

Preparation of samples and instrumentation for nanosecond transient absorption measurements were described previously (Byrdin et al. 2009; Gibasiewicz et al. 2013b). In brief, RC-only membranes were diluted to OD_800nm,1.5mm_ ≈ 0.5 in Tris–HCl buffer (pH 8.2) containing ~0.0001 % β-dodecyl maltoside (β-DM), ~50 % glycerol (v/v), 20 mM sodium ascorbate, and 12 mM *o*-phenanthroline. During the experiments, the samples were continuously illuminated with a white halogen light (~1 mW/cm^2^). Under these conditions, RCs are in the closed state with Q_A_ permanently reduced (Shuvalov and Parson 1981; Schenck et al. 1982; Gibasiewicz and Pajzderska 2008). *O*-phenanthroline is known to replace quinone *Q*_B_ thus preventing reoxidation of *Q*_A_^−^ by *Q*_B_ (Okamura et al. 1975; Michel et al. 1986). The samples were placed in a flat cuvette formed by two transparent round plastic plates separated by 1.5-mm rubber o-ring, and the cuvette was placed in a liquid nitrogen cryostat (Janis VPF-100) controlled by a temperature controller (LakeShore 331S-T2). The samples were excited at 532 nm (Nd:-YAG laser, Continuum Leopard SS-10), at a repetition rate of ~2 Hz, with light pulses of ~2 mJ energy and 100 ps duration. Monitoring light, directed orthogonally to the excitation beam, was generated at 690 nm by a laser diode (EOSI 2010) and detected by a fast photodiode (rise time 200 ps; model UPD-200-UP from Alphalas) connected to a digitizing oscilloscope (Agilent Infinium 81004B; 10 GHz; sampling rate, 40 G samples/s). The transient absorption signals were collected in a 6 μs temporal window and averaged over 1024 laser shots for each temperature. Experiments were performed at a range of temperatures between 77 and 298 K.

Kinetic traces were fitted with the sum of one or two exponential functions and a constant using Origin (OriginLab). The fitting was performed in a 100 and/or 200 ns window, and the starting point of the fits was at the maximum of the experimental kinetics.

### Femtosecond transient absorption measurements

Transient absorption measurements on the femtosecond time scale were performed at RT essentially as described earlier (Gibasiewicz et al. 2009) using the 1 kHz femtosecond laser system (Ti:sapphire, Spectra Physics) and a grating polychromator (Spectra Pro 150, Acton Research Corp.) with a thermoelectrically cooled CCD camera (Back Illumin., Princeton Instruments) described in detail previously (Maciejewski et al. 2000). The data were collected over a 2 ns temporal window and over a ~330–700 nm spectral window using ~300 fs excitation at 800 nm. RC-only membranes were diluted in either 15 mM Tris–HCl (pH 8.2) containing 0.025 % LDAO and 1 mM EDTA, or 50 mM glycine-NaOH (pH 10.5) containing 0.025 % β-DM. In order to keep the RCs in the open state (all electron transfer cofactors in their neutral state), fresh 10 mM sodium ascorbate was added to the sample before each experiment. In order to keep the RCs in the closed state (Q_A_ permanently reduced), 20 mM sodium ascorbate and 12 mM *o*-phenanthroline were added to the samples and continuous background illumination of the sample was applied during the measurement. Samples (~2.0 ml) were placed in a flat rotating quartz cuvette, allowing relaxation between each laser flash. The optical path of the probe laser beam in the sample was approximately 1.5 mm. White light probe pulses were generated in a calcium fluoride plate. Typically, absorption changes were measured at 70 different temporal points distributed unevenly between a few picoseconds before to two nanoseconds after the excitation pulse, with the pump-probe delay ranging from 100 fs around time zero to 200 ps at delays above 1000 ps. In the case of closed RCs, a part of measurements were performed for only 26 temporal points with lower resolution around time zero (pump-probe delays differences of 1 ps). This way, the experiments took less time that was essential for maintaining RCs in fully closed state all the time during the measurements.

The temporal evolution of the transient absorption spectra was subjected to global and target analysis (Holzwarth 1996; van Stokkum et al. 2004) using the software package Asufit developed at Arizona State University (available at http://www.public.asu.edu/~laserweb/asufit/asufit.html) as well as Glotaran software (Snellenburg et al. 2012). This evolution was fitted using sum of typically four exponential lifetimes convoluted with an instrumental response function modeled by a Gaussian of ~400 fs width.

## Results and discussion

### Nanosecond transient absorption measurements at 690 nm

**Fig. 3:**
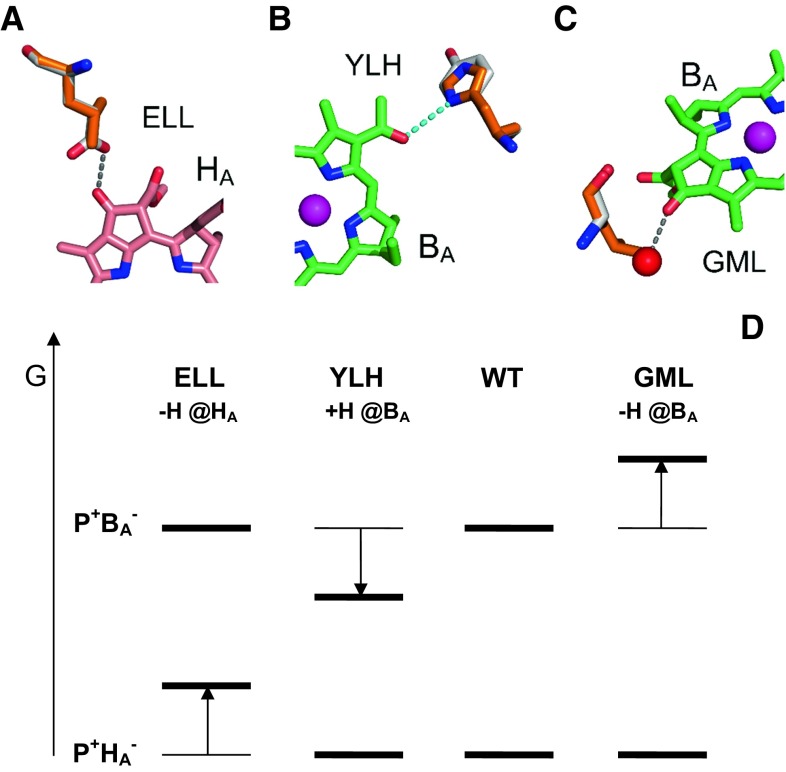
Single point mutations introduced to the mutant RC (**a**, **b**, **c**) and expected effects of these mutations on the free energy levels of the charge separated states caused by removal (labeled “−H”) or introduction (“+H”) of a hydrogen bond to either *H*_A_ or *B*_A_ (**d**). For *panels* (**a**–**c**), atom *colors* are as for Fig. 1. Carbons of the replaced amino acid are in white and carbons of the introduced amino acid are in orange. Lost hydrogen bonds are indicated with *gray dashed lines*, introduced hydrogen bonds with *cyan dashed line*. In (**c**) the *red sphere* is a hydrogen bond donor water molecule that is sterically excluded on replacing Gly with Leu

Nanosecond transient absorption spectroscopy was applied to antenna-deficient membranes containing either WT RCs or RCs with one of three single residue replacements near H_A_ or B_A_ that bring about known changes to the structure of the protein. Replacement of Glu L104 by Leu (ELL—Fig. 3a) and Gly M203 by Leu (GML—Fig. 3c) removed a hydrogen bond donor to *H*_A_ and *B*_A_, respectively (Bylina et al. 1988; Potter et al. 2005). Oppositely, replacement of Tyr L128 by His (YLH—Fig. 3b) provided a new hydrogen bond donor to *B*_A_ (Gibasiewicz et al. 2011). Figure 3d shows the expected effect of each mutation from the phenomenological rule that the removal of the hydrogen bond from a cofactor destabilizes (shifts up) the energy of the charge separated state involving this cofactor, whereas introducing a new hydrogen bond to a cofactor has the opposite effect (Lin et al. 1994). These mutant RCs, although widely characterized in many aspects, have not been studied with respect to the temperature dependence of charge recombination.

**Fig. 4:**
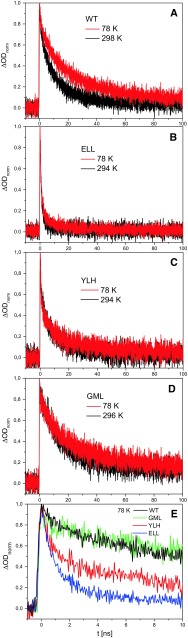
Comparison of absorption changes kinetics at 690 nm measured at RT and 78 K after picosecond excitation at 532 nm, revealing *P* ^+^ *H*_A_^−^ recombination

Figure 4 shows the kinetics of *P*^+^*H*_A_^−^ recombination measured as absorption changes at 690 nm for membrane-bound WT and mutant RCs. As was shown previously (Fajer et al. 1975; Heller et al. 1995; Huber et al. 1995; Gibasiewicz et al. 2009), changes in absorbance at this wavelength originate mostly from rapid (a few picoseconds) formation and slow (nanosecond) decay of the BPhe anion, *H*_A_^−^, as well as of the BChl anion, *B*_A_^−^. The relative contribution of the latter depends on the free energy gap between the states *P*^+^*H*_A_^−^ and *P*^+^*B*_A_^−^ (Kirmaier et al. 1991, 1995; Shkuropatov and Shuvalov 1993; Laporte et al. 1995; Heller et al. 1995, 1996; Schmidt et al. 1994, 1995; Huber et al. 1995; Arlt et al. 1996; Kennis et al. 1997) and may be negligible if the equilibrium is strongly shifted toward *P*^+^*H*_A_^−^. Any non-decaying signal on the 100 ns time scale is due to the formation of triplet states. As concluded previously (Gibasiewicz et al. 2013a), the relatively small amplitude of the non-decaying signal was due to a significantly smaller differential extinction coefficient of the triplet state relative to that of the *P*^+^*H*_A_^−^/*PH*_A_ transition at 690 nm (Volk et al. 1995). The transient absorption signal at 690 nm was fitted by a two-exponential function in each case except for the GML mutant for which a mono-exponential fit was performed. Parameters of the best fits are presented in Table 1.

**Table 1:** Fit parameters of charge recombination kinetics

	Sample	*T* (K)	Fit parameters^a^									
			Nanosecond experiment @ 690 nm						Femtosecond experiment @ 400–700 nm			
			*τ*_1_ (ns)	*A*_1_	*τ*_2_ (ns)	*A*_2_	*A*_0_	*τ*_av_^c^ (ns)	*τ*_1_ (ns)	*A*_1_	*τ*_2_ (ns) (fixed)	*A*_2_
2-exp	WT^b^	298	2.4	0.34	13	0.61	0.048	9.2	1.6	0.54	20	0.46
		78	3.8	0.29	26	0.62	0.093	18.9	–		–	
	ELL	294	1.0	0.92	20	0.07	0.007	2.3	0.68	0.77	20	0.23
		78	1.2	0.88	20	0.11	0.015	3.3	–		–	
	YLH	294	1.5	0.57	15	0.41	0.021	7.1	0.49	0.60	20	0.40
		78	1.2	0.59	20	0.35	0.053	8.1	–		–	
	GML	296	–	–	–	–	–	–	1.6	0.24	20	0.76
1-exp	GML	296	16.5	0.84	–	**–**	0.16	16.5	–	–	–	–
		78	23.7	0.80	–	**–**	0.20	23.7	–	–	–	–

^a^In the case of the nanosecond experiments, two- and one-exponential fits were performed according to the formula $\Delta A = \sum\nolimits_{i = 1}^{N} {A_{i} \exp ( - t/\tau_{i} ) + A_{0} }$ (*N* = 1 or 2) in a 200-ns temporal window (100-ns in the case of the WT RC) and the fitted kinetics are shown in Fig. 4. In the case of the femtosecond experiments, the lifetimes *τ*_1_ and *τ*_2_ come from the global analysis (Fig. 5), whereas the amplitudes *A*_1_ and *A*_2_ were taken from the target analysis (see Fig. 6) and are consistent with the relative amplitudes of the two phases of charge recombination shown in Fig. 5e–h

^b^Data taken from Gibasiewicz et al. (2013b)

^c^For two-exponential fits *τ*_av_ = (*τ*_1_ *A*_1_ + *τ*_2_ *A*_2_)/(*A*_1_ + *A*_2_). The estimated experimental error of the fit parameters obtained for the nanosecond experiment is ±20 %

Each of the mutations affected the overall kinetics (*τ*_ave_, Table 1), charge recombination being most rapid in the ELL mutant, least rapid in the GML mutant and with the YLH mutant being intermediate and closest to the WT. The differences were reflected by different lifetimes and relative amplitudes for the kinetic components (*τ*_1_, *τ*_2_, *A*_1_, and *A*_2_; Table 1). In addition, unlike for the WT RC where P^+^H_A_^−^ charge recombination was clearly temperature dependent, recombination in the mutant RCs was only weakly temperature dependent (Figs. 4b–d compared with Fig. 4a).

The kinetics of charge recombination in the WT, ELL, and YLH RCs (Fig. 4a–c) were well fitted by the two-exponential function predicted by the model shown in Fig. 2. In general, the model predicts a temperature-dependent overall decay of the *P*^+^*H*_A_^−^ state via the temperature-dependent lifetimes *τ*_T1_ and *τ*_T2_ (see Appendix in Supporting Information for the formulae). However, the weak temperature dependence of charge recombination for the two mutant RCs would still be consistent with this model if the initial (*P*^+^*H*_A_^−^)_1_ state was quasi-isoenergetic with *P*^+^*B*_A_^−^, whereas the relaxed state (*P*^+^*H*_A_^−^)_2_ was separated by a large free energy gap from *P*^+^*B*_A_^−^. It was estimated that for a τ_PB_ as short as 0.2 ns (Heller et al. 1996; Katilius et al. 1999), the energy gap of ~250 meV is large enough to make charge recombination from the relaxed state practically temperature independent (the recombination pathway via *P*^+^*B*_A_^−^ is inefficient, with *τ*_T2_ > 1000 ns both at RT and 77 K, and almost all recombination must occur via the direct, temperature-independent singlet or triplet pathway).

An alternative explanation for the weak temperature dependence of the *P*^+^*H*_A_^−^ charge recombination in the ELL and YLH RCs could be that the free energy gap between the states *P*^+^*H*_A_^−^ and *P*^+^*B*_A_^−^ is roughly proportional to the temperature. Thus, the lifetimes *τ*_T1_ and *τ*_T2_ remain almost temperature independent (Eqs. A21–A22 and A7–A8 in Supporting Information). This issue is treated in a quantitative way below (section: *Model*-*based estimation of the molecular parameters*) and in an extended way in Supporting Information.

In the case of the GML RCs, no clear fast phase of the order of 1–3 ns could be resolved in the nanosecond experiment (Fig. 4d). Instead, a mono-exponential fit yielded a lifetime of 16.5 ns at RT which increased to 23.7 ns at 78 K (Table 1). This increase suggests a possible minor contribution from a thermally activated pathway at RT or a temperature-dependent direct charge recombination.

The average decay lifetimes (*τ*_av_) both at RT and at 78 K for all four samples were well correlated with the amplitude (*A*_0_) of the non-decaying component assigned to triplet states. Across the four RCs, the slower the charge recombination the larger was the amount of triplet state.

### Model-based calculations of the molecular parameters

An extended description of the model-based calculations of molecular parameters shown in Fig. 2 can be found in Supporting Information. A summary of the main results is presented in Table 2. For WT, ELL, and YLH RCs, representative sets of molecular parameters estimated on the basis of the model parameters from single wavelength experiments (Table 1) are shown for the two cases introduced above. In case “*a*,” the free energy gaps ΔG_1_ and ΔG_2_ were fixed either at the values estimated on the basis of previous studies on WT RCs (Gibasiewicz et al. 2013b) or it was assumed that Δ*G*_1_ = 0 meV and Δ*G*_2_ = 250 meV (ELL and YLH RCs), the values for the mutants ensuring temperature independence of charge recombination as discussed above. In case “*b*,” Δ*G*_1_ and Δ*G*_2_ were free parameters. One can see that in the case *b*, the weak temperature dependence of charge recombination for the mutant RCs is ensured by decreased free energy gap between *P*^+^*H*_A_^−^ and *P*^+^*B*_A_^−^ at low temperatures (see the values of Δ*G*_1_ and Δ*G*_2_ at 78 K and at room temperature in Table 2). In this case, we assumed the same values of *τ*_PB_ as those estimated from the femtosecond experiment for WT, ELL, and YLH RCs (see below). Options *a* and *b* for the ELL RC are illustrated in Fig. S1 in the Supporting Information. The decay of GML mutant was not treated with the model shown in Fig. 2, since it was fitted by a single exponential component.

**Table 2:** Selected model molecular parameters for charge recombination kinetics

Sample	Nanosecond experiments						Femtosecond experiments		
	*T* (K)	Model parameters^a^					Model parameters (room temperature only)		
		Option	*τ*_12_ (ns)	Δ*G*_1_ (meV)	Δ*G*_2_ (meV)	*τ*_PB_ (ns)	*τ*_12_ (ns)	Δ*G*_1_ (meV)	*τ*_PB_ (ns)
WT	298	*a*	4.3 ± 1.1	*90 ± 18*	*128 ± 26*	*0.2*	–	–	–
		*b*	4.2 ± 1.1	77 ± 17	119 ± 14	*0.26 ± 0.15*	3.1 ± 0.2	60 ± 17	0.26 ± 0.15
	78	*a*	6.1 ± 1.5	*90 ± 18*	*128 ± 26*	*0.2*	–	–	–
		*b*	6.0 ± 1.5	25 ± 4	48 ± 16	*0.26 ± 0.15*			
ELL	296	*a*	6.5 ± 2.1	*0*	*250*	0.54 ± 0.05	–	–	–
		*b*	9.1 ± 3.7	22 ± 14	131 ± 14	*0.32 ± 0.10*	1.9 ± 0.4 (2.0 ± 0.3)	15 ± 15 (30 ± 15)	0.32 ± 0.10 (0.22 ± 0.10)
	78	*a*	5.0 ± 1.6	*0*	*250*	0.68 ± 0.13	–	–	–
		*b*	7.7 ± 2.8	7.9 ± 3.3	42 ± 10	*0.32 ± 0.10*			
YLH	296	*a*	1.9 ± 0.5	*0*	*250*	1.30 ± 0.29	–	–	–
		*b*	3.7 ± 1.0	79 ± 16	140 ± 16	*0.11 ± 0.07*	1.0 ± 0.1	49 ± 15	0.11 ± 0.07
	78	*a*	1.5 ± 0.4	*0*	*250*	0.97 ± 0.22	–	–	–
		*b*	2.9 ± 0.8	19 ± 4	50 ± 11	*0.11 ± 0.07*			
GML	RT	–	**–**	–	–	–	2.1	90	0.2

^a^Model parameters for the nanosecond experiments were estimated from the fit parameters of the nanosecond kinetics shown in Table 1 and the formulae shown in the Appendix (Supporting Information). Assumed values are shown in italics (in most cases, ranges of assumed values were considered). Uncertainties of the model parameters were estimated using the formula: $u\left( y \right) = \sqrt {\sum\nolimits_{i} {\left( {u\left( {x_{i} } \right) \frac{\partial y}{{\partial x_{i} }}} \right)^{2} } }$, where *y* is any of the model parameters, *x*_*i*_ is *i*-th independent variable (fit parameter), and $u\left( {x_{i} } \right)$ is the uncertainty of *i*-th independent variable (assumed to be ±20 %). Prior to uncertainty estimations, formulas for model parameters were transformed as a function of independent variables obtained directly from the fit, which were $A_{1} ,A_{2} ,A_{0} ,\tau_{1} ,\tau_{2}$. Options *a*—Δ*G*_1_ and Δ*G*_2_ values were assumed. Options *b*—Δ*G*_1_ and Δ*G*_2_ were free parameters. The two sets of model parameters for femtosecond experiments (ELL mutant) correspond to ways of estimation of Δ*G*_1_ depicted in Fig. 7 (the parameters in brackets correspond to Fig. 7a)

### Femtosecond transient absorption measurements from 400 to 700 nm

In order to verify the above considerations on the different sizes of the initial free energy gap between *P*^+^*B*_A_^−^ and the unrelaxed (*P*^+^*H*_A_^−^)_1_ state, RT femtosecond transient absorption measurements were performed over 2 ns and in a wide 400–700 nm spectral window allowing more precise identification of RC states and transitions between these states than is possible from single wavelength measurements. In addition, the better temporal resolution of femtosecond measurements enabled a more exact estimation of the fastest, subnanosecond phase of charge recombination (Gibasiewicz et al. 2009; Wang et al. 2012).

**Fig. 5:**
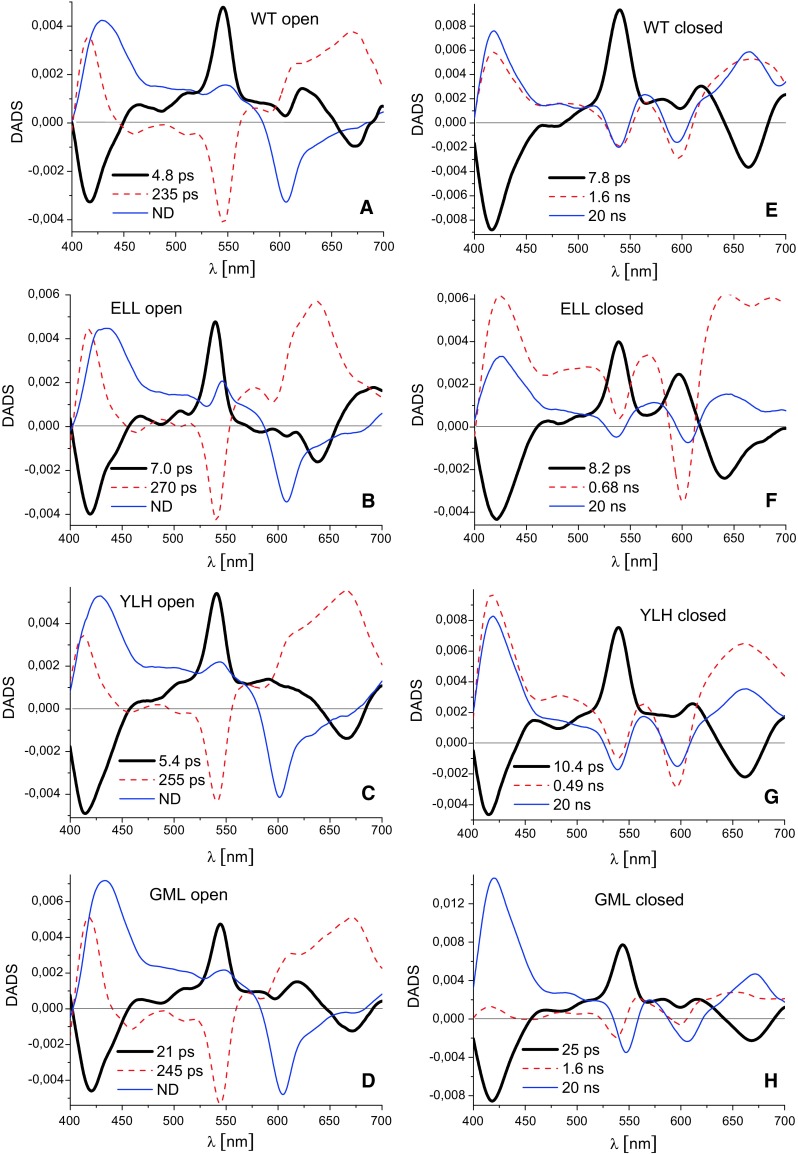
Decay-associated difference spectra for RCs in *open* and *closed* states. The spectra were estimated from femtosecond transient absorption difference spectra recorded following excitation at 800 nm with ~300 fs light pulses

Figure 5 compares the results of global fits of RT femtosecond data for the WT and mutant RCs in the form of decay-associated difference spectra (DADS) (Holzwarth 1996; van Stokkum et al. 2004). For each sample, DADS are shown for the open RCs in which there was free electron transfer from *H*_A_^−^ to *Q*_A_ (panels a–d), and for closed RCs in which the electron transfer from *H*_A_^−^ to *Q*_A_ was blocked (panels e–h). Where a DADS is positive, the absorption decreased in that spectral region within the specified lifetime, and where a DADS is negative the absorption increased with the respective lifetime.

### Charge separation in open RCs

Temporal evolution of transient absorption spectra for all four RCs was analyzed using three lifetimes and a non-decaying component. The fastest ~0.4 ps component was assigned to excitation energy transfer from the directly excited monomeric BChls at 800 nm to P (not shown). The remaining DADS for the WT membrane-bound RC (Fig. 5a) were similar to spectra published for isolated WT RCs (Gibasiewicz et al. 2009). The spectra obtained for the three mutant RCs (Fig. 5b–d) were generally very similar to those of the WT RC (Fig. 5A), as were the associated lifetimes (except for the GML RC, see below). The following does not describe precisely all details of the spectra, since this has been done previously (Gibasiewicz et al. 2009). Instead, we indicate minor differences between the DADS for the WT and mutant RCs and we focus on the spectral features that are most important for the further interpretation.

In each case (Fig. 5a–d), the black thick solid line is the amplitude spectrum of the charge separation reaction: P* → *P*^+^*H*_A_^−^. This reaction involves transient formation of the state *P*^+^*B*_A_^−^ but, due to its relatively slow formation and fast decay (~1 ps; see for example Arlt et al. 1993 and Parson and Warshel 2009 for reviews) its contribution is expected to be low and difficult to resolve. With the exception of the GML RC, charge separation was not strongly affected by the mutations and occurred within ~5–7 ps, a lifetime similar to that in the WT RC (Fig. 5a–c). The slower charge separation in the GML RC, ~21 ps, is consistent with a previous report (Potter et al. 2005). The charge separation DADS for all RCs showed a significant positive band at ~545 nm due to loss of H_A_ ground state absorption on formation of *H*_A_^−^. With the same lifetime, a new absorption band appeared as revealed by a negative band centered at ~670 nm, except for the ELL mutant for which this maximum was blue-shifted to ~640 nm. This band is assigned to *H*_A_^−^ but especially in the case of the ELL RC, it may also contain a contribution from the formation of *B*_A_^−^ (see below). The reason for the blue shift of this band in ELL RC is the removal of a hydrogen bond to *H*_A_ in this RC (Fig. 3a) (Bylina et al. 1988).

The main bands of the next DADS (red thin dashed line in Fig. 5a–d) were largely mirror reflections of the respective bands in the charge separation DADS. This was particularly the case for the bands at ~545 nm and at ~640/670 nm, assigned to electron transfer from *H*_A_^−^ to *Q*_A_. This is to be expected since charge separation populates *H*_A_^−^, whereas electron transfer from *H*_*A*_^−^ to *Q*_A_ recovers *H*_A_. The lifetime of the latter reaction was not significantly affected by the mutations (235 ps in the WT vs. ~250–270 ps in the mutant RCs). The non-decaying component (blue) originating from the long-lived state *P*^+^*Q*_A_^−^ was essentially identical for all RCs. DADS estimated for experimental data obtained at pH 10.5 (data not shown) were similar to those for data obtained at pH 8 (Fig. 5a–d).

### Charge separation in closed RCs

Standard experimental conditions commonly used to permanently reduce *Q*_A_ in isolated RCs were not fully effective in the case of membrane-bound RCs. The experiments performed under these conditions revealed the presence of mixed populations of open and closed RCs in the samples. It was established that increasing the buffer pH from 8 to 10.5 and shortening the time for the collection of each set of data at the expense of a decreased number of time points (thus leading to somewhat worse signal-to-noise ratio and lower temporal resolution) allowed the collection of data for fully or almost fully closed RCs (see below).

Charge separation DADS obtained for closed RCs at pH 10.5 were generally similar in lineshape to the respective DADS for open RCs except for the region around 600 nm in which, especially in the case of the ELL RCs, some differences were observed (compare panels a–d and e–h in Fig. 5). The lifetimes of charge separation were somewhat increased from ~5 to 7 ps in open RCs (Fig. 5a–c) to ~8–10 ps in closed RCs (Fig. 5e-g), and from 21 to 25 ps for the GML RC (Fig. 5h). This effect of pre-reduction of *Q*_A_ has been reported previously (Woodbury et al. 1985; Wang et al. 1994; Gibasiewicz et al. 2009). Changes in the spectral shape of the DADS for charge separation around 600 nm (wavelength of the *Q*_x_ absorption band of B_A_) may be rationalized by an increased contribution from *P*^+^*B*_A_^−^ admixing with *P*^+^*H*_A_^−^ (black spectrum) (Heller et al. 1996). In previous work on isolated WT RCs, an increase of the positive signal at ~600 nm after RC closure was shown and explained by a repulsive electrostatic interaction between *Q*_A_^−^ and *H*_A_^−^ that pushes the free energy of *P*^+^*H*_A_^−^ up toward that of *P*^+^*B*_A_^−^ (Gibasiewicz et al. 2009). Although in the present work, the spectral shape of the charge separation DADS at around 600 nm in open membrane-bound RCs is more complex than was reported previously for isolated RCs, careful comparison of the DADS for open and closed RCs reveals that the signal at around 600 nm is generally more positive for all the RCs after closing them except for the GML mutant (Fig. 5h). Therefore, we conclude that pre-reduction of *Q*_A_ leads in all the membrane-bound RCs under study (except for GML RC, see below) to a similar increase of the *P*^+^*H*_A_^−^ free energy level as reported for isolated RCs.

The effect of RC closure on the 600 nm region of the charge separation DADS was particularly prominent for the ELL RC (compare Fig. 5b and h). A prominent positive band appeared at this wavelength which was more than half as large as the *H*_A_/*H*_A_^−^ band at ~545 nm. We interpret this as a manifestation of substantial formation of *P*^+^*B*_A_^−^ in this RC due to a particularly large shift of the free energy of *P*^+^*H*_A_^−^ toward that of *P*^+^*B*_A_^−^ upon pre-reduction of *Q*_A_. In contrast, closure of the GML RC exerted almost no effect on the shape of the charge separation DADS in the ~600 nm region (Fig. 5d, h). This observation may mean that the interaction between *Q*_A_^−^ and *H*_A_^−^ does not result in an observable change in the initial free energy gap between the *P*^+^*B*_A_^−^ and *P*^+^*H*_A_^−^ in this RC.

### Charge recombination in closed RCs

Pre-reduction of *Q*_A_ causes back electron transfer from *H*_A_^−^ to *P*^+^ to be observed during the 2 ns measuring window. Therefore, the lineshapes of the two “slowest” DADS for closed RCs were markedly different from their equivalents for open RCs. Importantly, these closed RC DADS showed significant differences between RCs.

The two processes described by the two “slowest” DADS were, except for the GML RC, attributable to two phases of charge recombination (Fig. 5e–g; compare to data for isolated WT RCs in (Gibasiewicz et al. 2009)). In the case of GML RC, only the slowest phase could confidently be assigned to charge recombination (Fig. 5f). In principle, the phases of charge recombination should be the same as those observed in nanosecond measurements at 690 nm. However, given the different temporal resolutions, temporal windows and signal-to-noise ratios in these two types of experiment, the lifetimes resolved were somewhat different. As expected, the lifetimes of the fast phase of charge recombination, *τ*_1_, were found to be smaller in the femtosecond experiments (see comparison of *τ*_1_ values in nanosecond and femtosecond experiments in Table 1). Importantly, however, results from both techniques showed that the fast phase of charge recombination was slower in WT RCs than in the ELL and YLH mutants, suggesting a larger energetic barrier for this recombination in the former. The 1.6-ns component resolved in the femtosecond experiment for the GML RC had no counterpart in the nanosecond experiment, probably due to its small amplitude at 690 nm and the poorer signal-to-noise ratio in the nanosecond experiment.

The temporal window of 2 ns in the femtosecond experiment was insufficient for a proper estimation of the lifetimes of the slow charge recombination phase. Therefore, in analyzing the data from the femtosecond experiment, the lifetime of the slow phase was fixed for all RCs at 20 ns. As expected and checked, the exact value of this parameter was not essential for the results of fitting procedure.

It is interesting to note the differences between the relative amplitudes of the “slow” and “fast” charge recombination DADS. Focusing on the spectral region covering the bands at ~545 and ~600 nm, it can easily be seen that in the case of WT and YLH RCs (Fig. 5e, g), the contributions of both phases were roughly equal, whereas in the case of ELL RC, the contribution of the 0.68 ns component was much larger than that of the 20 ns component (Fig. 5f). These observations correlate very well with the relative amplitudes from the nanosecond fits (Table 1).

The shape and relative amplitude of the bands in the fast nanosecond DADS found for the GML RC (1.6 ns; Fig. 5h) were markedly different from those obtained for the remaining RCs. The main features of this DADS was “differential-like” structures at ~545 and ~600 nm suggesting that the process underlying this 1.6 ns DADS is not primarily the fast phase of charge recombination as it is in the remaining RCs but rather an electrochromic shift of BPhe absorbance at ~545 nm and BChl absorbance at ~600 nm. This hypothesis was confirmed through a target analysis of the femtosecond data.

### Target analysis

**Fig. 6:**
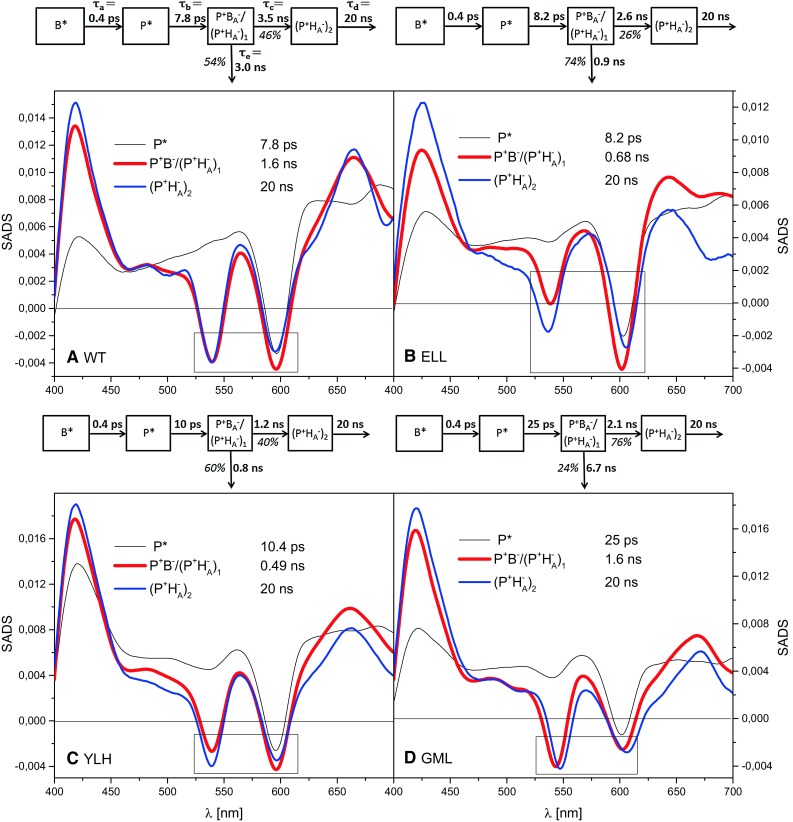
Species-associated difference spectra and compartmental model schemes for RCs in closed state. The spectra were estimated from femtosecond transient absorption difference spectra recorded following excitation at 800 nm with ~300 fs light pulses. Reversibility of the reaction *P* ^+^ *B*_A_^−^/(*P* ^+^ *H*_A_^−^)_1_ → (*P* ^+^ *H*_A_^−^ )_2_ was ignored in the modeling due to large free energy gap between the states *P* ^+^ *B*_A_^−^/(*P* ^+^ *H*_A_^−^)_1_ and (*P* ^+^ *H*_A_^−^)_2_. The five lifetimes obtained from the target analysis yields the four apparent lifetimes obtained from the global analysis

Figure 6 presents results of target analysis performed for the femtosecond data collected for closed RCs. A common model of considered states was used for all samples consisting of the following four compartments or species: a BChl excited state (B*), the excited state of *P* (P*), a charge separated state equilibrated over *P*^+^*B*_A_^−^ and an unrelaxed (*P*^+^*H*_A_^−^)_1_ state, and a relaxed (*P*^+^*H*_A_^−^)_2_ charge separated state (see the schemes above the spectra in Fig. 6). The lifetimes of 0.4 ps (B* → P*) and 20 ns (depicting (*P*^+^*H*_A_^−^)_2_ charge recombination) were fixed. Moreover, a constraint was applied that the sum of amplitudes of the bands at ~545 and ~600 nm for the fast charge recombination spectrum should be the same as the sum of the amplitudes of the respective bands for the slow charge recombination spectrum. (The amplitudes of these bands were measured relative to the local maximum at ~565 nm). This constraint was justified by an assumption that the differential molar extinction coefficients related to photobleaching signals from *H*_A_^−^ at ~545 nm are the same as those from *P*^+^ and *B*_A_^−^ at ~600 nm (see below). The resulting spectra of the excited state P* (black thin lines in Fig. 6) are rather similar to each other for the four samples. In the analysis, one should focus on the differences in the relative amplitudes of the bands at ~545 and ~600 nm in fast (thick red lines) and slow (blue lines) charge recombination spectra.

As mentioned in the Introduction, the relative amplitudes of the Q_x_ photobleaching bands at ~545 and ~600 nm in the two charge recombination phases are good indicators of the free energy gap between *P*^+^*B*_A_^−^ and *P*^+^*H*_A_^−^ on the respective time scales. The bigger the signal at ~600 nm relative to that at ~545 nm, the smaller this gap and the more the *P*^+^*B*_A_^−^ ⟷ *P*^+^*H*_A_^−^ equilibrium is shifted toward the left. Comparison of these two negative bands in the fast recombination species-associated difference spectra (SADS; Fig. 6a–c, red; 1.6 ns for WT, 0.68 ns for ELL, and 0.49 ns for YLH) clearly shows that for WT and YLH RCs, the band at ~600 nm is only slightly deeper than that at ~545 nm, whereas in ELL RC the difference between these bands is much bigger. This difference is consistent with the postulate formulated above that the free energy gap between *P*^+^*B*_A_^−^ and unrelaxed state (*P*^+^*H*_A_^−^)_1_, is smaller for ELL than for the WT and YLH RCs.

**Fig. 7:**
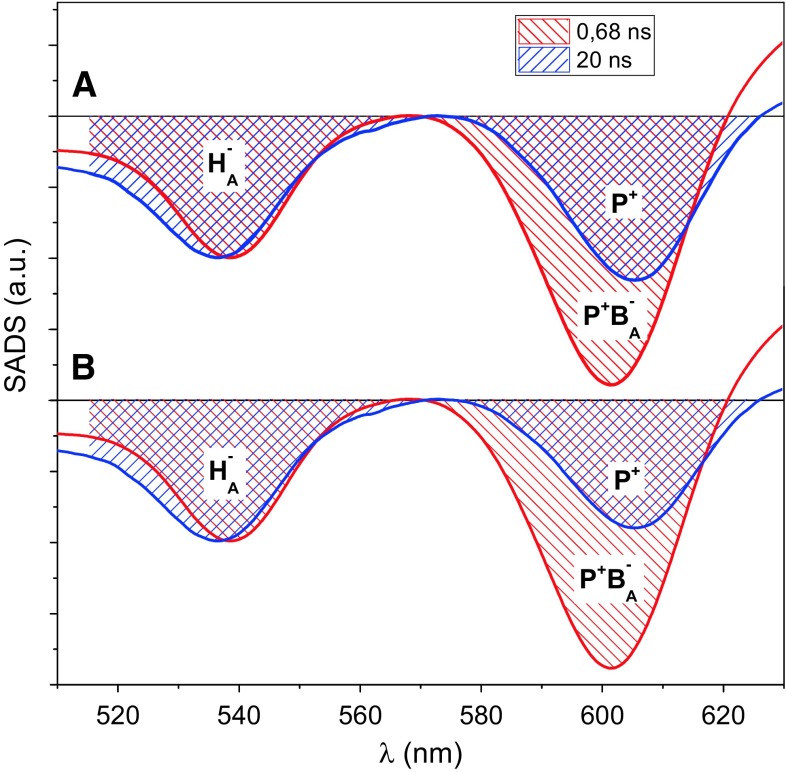
Comparison of the “fast” and “slow” charge recombination SADS of ELL RCs in the spectral region of Q_x_ bands at ~545 and ~600 nm. The original SADS (from Fig. 6b) were vertically shifted relative to one another and normalized in order to have the same amplitudes at ~565–570 and ~540 nm. This way, the area above the ~600 nm band of the “slow” SADS represents the contribution of *P* ^+^ from the state *P* ^+^ *H*_A_^−^ in the ~600 nm band of the “fast” SADS, whereas the remaining area of the latter band represents the contributions of *P* ^+^ and *B*_A_^−^ from the state *P* ^+^ *B*_A_^−^. The respective areas labeled “*P* ^+^” and “*P* ^+^ *B*_A_^−^” allows estimation of the free energy gap between unrelaxed *P* ^+^ *H*_A_^−^ and *P* ^+^ *B*_A_^−^ from the equation: $\Delta G = kT\ln \frac{``{P^{+} }"}{{``{P^{+} B_{\text{A}}^{-}"}}}\frac{{\Delta \varepsilon (P^{ + } - P)}}{{\Delta \varepsilon \left( {P^{ + } - P} \right) + \Delta \varepsilon \left( {B_{\text{A}}^{ - } - B_{\text{A}} } \right)}}$, where *k* is the Boltzmann constant, *T*—absolute temperature, Δ*ε*(*P* ^+^ − *P*), and Δ*ε*(*B*_A_^−^ − *B*_A_)—mean differential absorption coefficients of the respective species within the 600-nm band. **a**—the “slow” charge recombination SADS was taken directly from the target analysis shown in Fig. 6b. **b**–the “slow” charge recombination SADS was corrected in the following way. It was assumed that a part of the ~600-nm band was due to a contribution from open RCs; in order to reject this contribution, the amplitude of the original ~600-nm band was vertically shrunk in such a way that the resulting amplitude ratio of the bands at ~545 and ~600 nm was the same as for WT RCs (compare to the “slow” charge recombination SADS in Fig. 6b)

The relative amplitudes of the bands at ~545 and ~600 nm in the “slow” charge recombination spectrum were shown in a previous publication on isolated WT RCs to be similar to each other, with the band at ~545 nm being slightly deeper (Gibasiewicz et al. 2009). A similar pattern is observed for WT, YLH, and GML membrane-bound RCs (Figs. 6a,c,d, blue). An opposite observation that the band at ~600 nm was somewhat deeper than that at ~545 nm in the case of the ELL RC (Fig. 6b, blue) may be explained by a minor admixture of RCs in the open state. This explanation seems to be very likely since we found that full closing of RCs was difficult for all samples—it was necessary to increase pH of the RCs solution to 10.5 in order to close RCs efficiently. However, we also considered an alternative possibility that the band at 545 nm is intrinsically smaller than that at 600 nm in the case of the ELL RC, due to the mutation which may affect *Q*_*x*_ band of *H*_A_. Apparently, the mutation affects also the absorption band of *H*_A_^−^ above 625 nm which is clearly blue-shifted compared to the remaining samples (compare “fast” and “slow” charge recombination SADS in this region for all samples (Fig. 6)). The two approaches result in somewhat different estimations of the free energy gap between *P*^+^*B*_A_^−^ and unrelaxed *P*^+^*H*_A_^−^ (see Fig. 7 and below). For all samples, the “slow” charge recombination SADS is believed to originate from the relaxed state of *P*^+^*H*_A_^−^ (*P*^+^*H*_A_^−^)_2_, with a negligible contribution from *P*^+^*B*_A_^−^ in analogy to the slow phase obtained in the nanosecond experiments.

The relative amplitudes or areas of the photobleaching bands at ~545 and ~600 nm in the charge recombination SADS (Fig. 6a–c) may serve to estimate the relative populations of the equilibrated states *P*^+^*B*_A_^−^ and (*P*^+^*H*_A_^−^)_1_ as well as the free energy gap between these states in a quantitative way described previously (Gibasiewicz et al. 2009) and presented in Fig. 7 for the case of the ELL RCs. In brief, assuming that the 20 ns SADS is contributed to exclusively by relaxed state (*P*^+^*H*_A_^−^)_2_ (with the band at ~545 nm assigned to H_A_^−^ and the band at ~600 nm assigned to *P*^+^), the “excess” of the photobleaching signal of the ~600 nm band in the fast charge recombination SADS (0.49–1.6 ns) relative to the 20 ns SADS in each dataset (except for GML RCs) is a measure of the population of *P*^+^*B*_A_^−^ that is in equilibrium with (*P*^+^*H*_A_^−^)_1_. Assuming further similar differential extinction coefficients of the photobleaching signals, Δε, for P^+^/P and *B*_A_^−^/*B*_A_ at ~600 nm (0.5Δ*ε*(*P*^+^ − *P*) < Δ*ε*(*B*_A_^−^ − *B*_A_) < 2Δ*ε*(*P*^+^ − *P*)) and testing somewhat different ways of estimation of the photobleaching bands’ areas at ~545 and ~600 nm (one of them is illustrated in Fig. 7; in another, we limited the integration of the band to the region of the band minimum (~545 or ~600 nm) ± 20 nm), a set Δ*G*_1_ of ranges was obtained (Table 2).

For the GML RC, unlike for the remaining complexes, the relative amplitudes of the bands at ~545 and ~600 nm in the fast charge recombination SADS were very similar to the corresponding amplitudes in the slow charge recombination SADS (Fig. 6d, red and blue). This observation indicates that the contribution of the state *P*^+^*B*_A_^−^ being in equilibrium with (P^+^H_A_^−^)_1_ is negligible and that the initial free energy gap between these states (Δ*G*_1_) is large, which is confirmed by calculations (Table 2, Δ*G*_1_ = 90 meV for the assumed *τ*_PB_ value of 0.2 ns). On the other hand, the two negative bands at ~545 and ~600 nm in the slow charge recombination SADS are red shifted relative to those in the fast charge recombination SADS. This spectral shift explains the “differential-like” shape of the 1.6-ns DADS (Fig. 5h).

The lifetimes estimated from the target analysis (*τ*_a_ − *τ*_e_) are presented in Fig. 6 in the compartmental model schemes. They allow direct estimation of the branching ratio that determines what percentage of the RCs in equilibrated state *P*^+^*B*_A_^−^/(*P*^+^*H*_A_^−^)_1_ recombine (with lifetime *τ*_e_) and what percentage evolve to the relaxed state (*P*^+^*H*_A_^−^)_2_ (with lifetime *τ*_c_)—values are given in the schemes (Fig. 6). Moreover, together with the values of Δ*G*_1_ estimated from the same femtosecond experiment (Table 2), the lifetimes obtained from the target analysis (*τ*_c_ and *τ*_e_) allow independent estimation of the molecular lifetimes *τ*_12_ and *τ*_PB_ (Fig. 2) from the following formulae:

$$\tau_{12} = P_{1} \tau_{\text{c}} = \frac{{e^{{\Delta G_{1} /kT}} }}{{1 + e^{{\Delta G_{1} /kT}} }}\tau_{\text{c}} ,$$

$$\tau_{\text{PB}} = (1 - P_{1} )\tau_{\text{e}} = \frac{1}{{1 + e^{{\Delta G_{1} /kT}} }}\tau_{\text{d}},$$

where $(1 - P_{1} )$ and $P_{1}$are the probabilities of occupation of the states *P*^+^*B*_A_^−^ and (*P*^+^*H*_A_^−^)_1_, respectively, in the *P*^+^*B*_A_^−^/(*P*^+^*H*_A_^−^)_1_ equilibrium, *k* is the Boltzmann constant, and *T* is the absolute temperature.

### Comparative analysis of the results obtained from the nanosecond and femtosecond experiments

The nanosecond and femtosecond measurements gave consistent raw results, although both the relative amplitudes *A*_1_–*A*_2_ and lifetimes *τ*_1_–*τ*_2_ of the two charge recombination phases obtained from the two techniques were somewhat different (Table 1). In the case of the femtosecond measurements, the amplitudes of the two phases were similar to one another for the WT RC, with the fast phase dominating over the slow phase to a moderate extent for the YLH RC or to a large extent for the ELL RC. The slow phase largely dominated over the fast phase for the GML RC (see also Fig. 5e–h). Similar tendency in *A*_1_/*A*_2_ proportions were observed in the nanosecond experiments (Table 1). Some differences may be explained by different temporal resolution and temporal windows in these two techniques, as well as a worse signal-to-noise ratio in the nanosecond experiment. Probably for the same reasons, no fast component was resolved for GML RC in the nanosecond experiment.

Analysis of the model molecular parameters obtained from the nanosecond and femtosecond data (Table 2) leads to the following observations. Treating the free energy gaps as free parameters (case *b* in the nanosecond experiment), both techniques indicate similar initial free energy gaps (Δ*G*_1_) between the states *P*^+^*B*_A_^−^ and (*P*^+^*H*_A_^−^)_1_ for WT (77 and 60 meV), ELL (22 and 15 meV), and YLH RCs (79 and 49 meV). The significant value of Δ*G*_1_ for the YLH RC estimated from the femtosecond experiment seems to exclude the possibility that the weak temperature dependence of charge recombination is caused by isoenergeticity of *P*^+^*B*_A_^−^ and (*P*^+^*H*_A_^−^)_1_ in this case (option *a*). Oppositely, the small value of Δ*G*_1_ for the ELL RC makes both options (*a* and *b*) likely. According to both femtosecond and nanosecond results, all three molecular parameters shown in Table 2, *τ*_12_, Δ*G*_1_, and *τ*_PB_, co-determine the overall charge recombination kinetics. The femtosecond results, characterized by better temporal resolution, indicate that the protein relaxation is accelerated in all three mutants relative to WT RC, with this relaxation being particularly fast for YLH RC, 1 ns versus 3.1 ns for WT. Ranges of the *τ*_PB_ values extracted from the femtosecond experiments (from 0.11 ns for YLH to 0.32 ns for ELL) agree well with those published before (Heller et al. 1996; Katilius et al. 1999).

## Conclusions

The observed variety of charge recombination dynamics in the mutant RCs is caused by three factors undergoing modulation by the introduced mutations: (1) the initial free energy gap between the states *P*^+^*B*_A_^−^ and *P*^+^*H*_A_^−^, (2) the intrinsic rate of *P*^+^*B*_A_^−^ → *PB*_A_ charge recombination, and (3) the dynamics of protein relaxation in response to the appearance of the charge separated states. The main factor responsible for the particularly fast charge recombination in the ELL RC is small initial free energy gap between *P*^+^*B*_A_^−^ and *P*^+^*H*_A_^−^, whereas large initial free energy gap between these two states is responsible for particularly slow charge recombination in the GML mutant. The YLH RC showed a similar average decay of the charge separated states to that of WT RCs, despite a particularly fast *P*^+^*B*_A_^−^ → *PB*_A_ charge recombination which was compensated for by an increased efficiency of protein relaxation. In all the mutant RCs, no significant temperature dependence of the protein relaxation could be concluded from the model calculations, in line with a previous report on WT RCs. The question of whether the free energy gap between *P*^+^*B*_A_^−^ and *P*^+^*H*_A_^−^ states is temperature dependent or not was not definitely answered for the WT, ELL, and GML RCs. Model calculations for YLH RCs indicate that this free energy gap decreases at low temperature but still undergoes the temporal evolution. An identification of the structural rearrangements underlying the mutation-specific variations in protein dynamics is a real challenge and will be the subject of future work.

## Electronic supplementary material

Below is the link to the electronic supplementary material.

Supplementary material 1 (DOCX 95 kb)
