# Supplementary material for: Weak temperature dependence of P+HA− recombination in mutant Rhodobacter sphaeroides reaction centers
Source: Photosynth Res. 2016 Mar 4;128:243–58. doi: 10.1007/s11120-016-0239-9 (PMC4877430; doi:10.1007/s11120-016-0239-9)
Supplement: Supplementary file 1 — Supplementary material 1 (DOCX 95 kb) [file 11120_2016_239_MOESM1_ESM.docx]

**Supporting Information**

*Extended description of the model-based calculations of the molecular parameters*

The observed weak temperature dependence of the charge recombination reaction in the ELL and YLH RCs may be explained in two ways using the model shown in Fig. 2 and data from the nanosecond measurements. The first assumes that the initial charge separated state (P^+^H_A_^-^)_1_ is quasi-isoenergetic with P^+^B_A_^-^ and the relaxed charge separated state (P^+^H_A_^-^)_2_ is energetically far below P^+^B_A_^-^ (option “*a”*). The second assumes that the free energy gaps between P^+^B_A_^-^ and (P^+^H_A_^-^)_1_ and between (P^+^H_A_^-^)_1_ and (P^+^H_A_^-^)_2_ may decrease with decreasing temperature, which causes a similar efficiency of thermally activated charge recombination, via P^+^B_A_^-^, at different temperatures (option “*b”*; see below). Other words, lowering the thermal energy is compensated by lowering the energetic barrier of the charge recombination reaction. Both of these options are discussed separately below. For WT RCs two subcases with fixed ΔG_i_ were considered – one with τ_PB_ fixed at the level of 0.2 ns (option *a*) and another one with τ_PB_ =1 ns (option *a’*).

*Option a: Constraints on values of ΔG_i_*. On the basis of the parameters from the fits of the nanosecond kinetic data (Table 1 and Fig. 4) the molecular parameters shown in the model in Fig. 2 can be estimated. In the following, instead of the lifetimes shown in Fig. 2 their reciprocals, rate constants (k) are used. In the Appendix, formulae relating the fit and molecular parameters are presented. Unfortunately, the number of four linearly independent equations, A5, A6, A15, and A16 (see Appendix), resulting from solution of the proper set of differential equations (Eqs. A1) is lower than the number of seven molecular parameters ($k_{12},k_{t},k_{dir1},k_{dir2},k_{\mathrm{PB}},\Delta G_{1},\Delta G_{2}$), resulting from the model (Fig. 2). Therefore, it is necessary to make assumptions as to the values of some of these parameters in order to estimate the remaining ones. Obviously, this may be done in a multiple ways. However, given the qualitative considerations presented above, it seems justifiable to assume first that for the ELL and YLH RCs, ΔG_1_ = 0 meV and ΔG_2_ = 250 meV. As mentioned above, the precise value of ΔG_2_ is not essential for the estimations, the essential factor being that it is sufficiently large that thermally activated charge recombination from the relaxed P^+^H_A_^-^ state is not effective and thus may be neglected. The above assumptions regarding the values of ΔG_1_ and ΔG_2_ are sufficient to yield the weak or absent temperature dependence of charge recombination in the mutant RCs. An additional assumption for the ELL and YLH RCs is that k_dir1_ = k_dir2_.

As can be seen from the formulae in the Appendix (Eqs. A20-A21), the relaxation and triplet parameters, k_12_ and k_t_, may be estimated directly from the fit parameters (Table 1) with the only assumption made on the values of ΔG_1_ and ΔG_2_ via the population factors P_1_ and P_2_ (Eqs. A7-A8) describing the probability that the state P^+^H_A_^-^, being in equilibrium with P^+^B_A_^-^, is populated. It should be noted that P_1_ = 1/2 for ΔG_1_ = 0 meV and P_2_ ≈ 1 for ΔG_2_ = 250 meV, independent of temperature.

Values of τ_12_ and τ_t_ estimated for the mutant RCs using the above constraints on ΔG_i_ are collected in Table S1 (option *a*). In the case of the WT RC the values of ΔG_1_ and ΔG_2_ were fixed on temperature-independent levels of 90 and 128 meV (±20%), respectively. These ΔG_i_ values were obtained previously (Gibasiewicz et al. 2013b) from the temperature dependence of the fit parameters τ_1_ and τ_2_ (defined as those shown in Table 1), under the assumption that the sole source of their temperature dependence is decreasing accessibility of the state P^+^B_A_^-^ when lowering the temperature. In the present model, the values of ΔG_i_ for the WT RC depend strongly on τ_PB_ and in option *a* they were estimated assuming τ_PB_ = 0.2 ns (Heller et al. 1996; Katilius et al. 1999). The only major effect of increasing τ_PB_ to 1 ns (Schmidt et al. 1994; Kirmaier et al. 1995) was, as expected, decreasing the values of ΔG_1_ and ΔG_2_ to 44 and 85 meV, respectively (Table S1; option *a’*).

It can be seen that the relaxation lifetime τ_12_ for the ELL RC is 5 ns at 78 K and 6.5 ns at 296 K, not very different from equivalent values estimated for WT RCs 6.1 ns at 78 K and ~4 ns at 298 K). Under the above constraints, the much faster overall charge recombination in the case of the ELL RC is caused by the isoenergetic character of the states P^+^B_A_^-^ and (P^+^H_A_^-^)_1_ and, consequently, by particularly efficient charge recombination via P^+^B_A_^-^ and not by particularly slow relaxation, which in principle could also contribute to acceleration of charge recombination.

RCs of the YLH mutant are characterized by an approximately three times faster protein relaxation (τ_12_ = 1.5 ns at 78 K and 1.9 ns at 294 K; Table S1; option *a*) than in ELL RCs despite the fact that (P^+^H_A_^-^)_1_ was assumed to be isoenergetic with P^+^B_A_^-^ in both RCs. The fast relaxation is one of the reasons why the relative amplitude of the slower phase is significantly larger in the YLH RC than in the ELL RC; simply relaxation to the state (P^+^H_A_^-^)_2_ is a more competitive pathway in the YLH RC (Fig. 2). Another reason could in principle be the slower intrinsic charge recombination lifetime, τ_PB_, from P^+^B_A_^-^ in the YLH RC (Table S1), although this is not confirmed by the femtosecond experiment (see the main text).

Formation of a triplet state in all the RCs undergoing the modelling was characterized by a lifetime τ_t_ ranging from ~30 to ~60 ns which was somewhat temperature-dependent, but not sensitive to different constraints put on the model parameters (Table S1; options *a* and *b*).

Table S1 also shows lifetimes τ_dir1_, τ_dir2_, and τ_PB_ calculated from Eqs. A5 and A6 under additional constraints (τ_dir1_ = τ_dir2_ for the ELL and YLH RCs, as described above). However, it should be noted that the constraint τ_dir1_ = τ_dir2_ is not necessarily valid. We have also assumed one single value for τ_PB_ which is also not necessarily valid. The value of τ_PB_ may be different in unrelaxed and relaxed states of the RC, and therefore the values of the parameters τ_dir1_, τ_dir2_, and τ_PB_ shown in Table S1 should be treated as very approximate.

*Option b: Constraints on values of τ_diri_ and τ_PB_*. Leaving ΔG_1_ and ΔG_2_ as free parameters and fixing τ_dir_*_i_* and τ_PB_ led to an alternative interpretation of the weak temperature dependence of charge recombination observed in the ELL and YLH RCs. Under such conditions the energy levels of the states (P^+^H_A_^-^)_1_ and (P^+^H_A_^-^)_2_ become temperature dependent. Table S1 shows results of estimations performed for values of τ_PB_ taken from the modelling of the femtosecond data (see the main text). It was further assumed that τ_dir1_ = τ_dir2_ = 100 ns (smaller values of τ_dir1_ = τ_dir2_ led to non-physical solutions). With these assumptions, for ELL RC the free energy of (P^+^H_A_^-^)_1_ remained almost isoenergetic with P^+^B_A_^-^, independent of temperature, whereas the energy of (P^+^H_A_^-^)_2_ strongly depended on temperature (ΔG_2_ equals 131 meV at RT and only 42 meV at 78 K). For the YLH RC both ΔG_1_ and ΔG_2_ significantly changes with temperature. The free energy levels estimated for the ELL RC (options *a* and *b*) are presented in Fig. S1. Interestingly, in option *b* the estimated relaxation lifetimes, τ_12_, are not significantly different from those estimated in option *a*.

The assumed constraints under option *b* were also applied to WT RCs (Table S1). It turned out that the strong temperature dependence of charge recombination in WT RCs may be modeled not only under the assumption of temperature-independence of ΔG_1_ and ΔG_2_ as was done previously (Gibasiewicz et al. 2013b) but also when treating these gaps as free parameters. The results presented in Table S1 show that similar to the case for the YLH RCs, the free energy gaps ΔG_1_ and ΔG_2_ in the WT RC decrease with decreasing temperature, although the effect is less pronounced as expected from stronger temperature dependence of charge recombination in WT RCs.

**Appendix**

In order to relate molecular parameters shown in Fig. 2 with the fit parameters obtained experimentally in the nanosecond experiment and presented in Table 1 one needs to solve the following set of differential equations (with lifetimes replaced by their reciprocals – rate constants; compare very similar models used before (Gibasiewicz et al. 2013a, 2013b)):

$\left\{ \begin{aligned} \frac{dA(t)}{\mathrm{dt}}=-k_{PH1}A\left( t \right) \\ \frac{\mathrm{dB}\left( t \right)}{\mathrm{dt}}=k_{12}P_{1}A\left( t \right)-k_{PH2}B\left( t \right) \\ \frac{\mathrm{dC}\left( t \right)}{\mathrm{dt}}=k_{t}P_{2}B\left( t \right) \end{aligned} \right.$ (A1)

where *t* stands for time,

$A\left( t \right)=\left[ \left( P^{+}H_{A}^{-} \right)_{1} \right]\left( t \right)+[\left( P^{+}B_{A}^{-} \right)_{1}](t)$ (A2)
$B\left( t \right)=\left[ \left( P^{+}H_{A}^{-} \right)_{2} \right]\left( t \right)+[\left( P^{+}B_{A}^{-} \right)_{2}](t)$ (A3)

C(t) = [^3^P](t) (A4)

and $\left[ \left( P^{+}H_{A}^{-} \right)_{i} \right]\left( t \right) and [\left( P^{+}B_{A}^{-} \right)_{i}](t)$ (i = 1, 2) are transient concentrations of the respective states shown in Fig. 2. (P^+^B_A_^-^)_1_ and (P^+^B_A_^-^)_2_ are the states being in equilibrium with the states (P^+^H_A_^-^)_1_ and (P^+^H_A_^-^)_2_, respectively; in the energetic scheme, the states (P^+^B_A_^-^)_1_ and (P^+^B_A_^-^)_2_ are drawn as having identical energy levels and are labeled P^+^B_A_^-^. This is because the aim of the Fig. 2 is to show energetic relaxation of the state P^+^H_A_^-^ relative to that of the state P^+^B_A_^-^. However, it is likely that the state P^+^B_A_^-^ relaxes in parallel with the state (P^+^H_A_^-^) but to a different extent.

Furthermore,

$k_{PH1}=k_{\mathrm{PB}}\left( 1-P_{1} \right)+\left( k_{12}+k_{dir1} \right)P_{1}$ (A5)
$k_{PH2}=k_{\mathrm{PB}}\left( 1-P_{2} \right)+\left( k_{t}+k_{dir2} \right)P_{2}$ (A6)

where P_1_ and P_2_ are probabilities of population of the states (P^+^H_A_^-^)_1_ and (P^+^H_A_^-^)_2_, respectively, being in equilibrium with the respective states of P^+^B_A_^-^ (the total probability of population of the (P^+^H_A_^-^)_1_ and (P^+^B_A_^-^)_1_ states is normalized to one; similarly for the (P^+^H_A_^-^)_2_ and (P^+^B_A_^-^)_2_ states):

$P_{1}=\left( 1+\exp\left( -\frac{\Delta G_{1}}{\mathrm{kT}} \right) \right)^{-1}$ (A7)
$P_{2}=\left( 1+\exp\left( -\frac{\Delta G_{2}}{\mathrm{kT}} \right) \right)^{-1}$ (A8)

Solution of the set of differential equations leads to the following formulae:

$A\left( t \right)=ⅇ^{-k_{PH1}t}$ (A9)
$B\left( t \right)=-\frac{\left( ⅇ^{-k_{PH1}t}-ⅇ^{-k_{PH2}t} \right)k_{12}P_{1}}{k_{PH1}-k_{PH2}}$ (A10)$C\left( t \right)=\frac{k_{12}\left( k_{PH1}-ⅇ^{-k_{PH2}t}k_{PH1}+\left( -1+ⅇ^{-k_{PH1}t} \right)k_{PH2} \right)k_{t}P_{1}P_{2}}{k_{PH1}\left( k_{PH1}-k_{PH2} \right)k_{PH2}}$ (A11)

These equations describe concentrations of different states in the system. In order to get a formula for change of absorbance one has to use differential extinction coefficients for different states. In general:

$\Delta\mathrm{OD}\left( t \right)=A\left( t \right)\cdot\Delta\varepsilon\left( P^{+}H_{A}^{-}/PH_{A} \right)+B\left( t \right)\cdot\Delta\varepsilon(P^{+}H_{A}^{-}/PH_{A})+C\left( t \right)\cdot\Delta\varepsilon(^{3}P/P)$ (A12)

Assuming, as before (Gibasiewicz et al. 2013a, 2013b), that at 690 nm $\Delta\varepsilon\left( P^{+}H_{A}^{-}/PH_{A} \right)=\Delta\varepsilon\left( P^{+}B_{A}^{-}/PB_{A} \right)=5\Delta\varepsilon(^{3}P/P)$ the formula for normalized differential absorbance is given by:

$\Delta\mathrm{OD}\left( t \right)=A\left( t \right)+B\left( t \right)+\frac{1}{5}C\left( t \right)$ (A13)

After substitution of A9-A11 to A13 one obtains an equation identical to that one used in two-exponential fitting of the nanosecond kinetics:

$\Delta\mathrm{OD}\left( t \right)=A_{1}\exp\left( -\frac{t}{\tau_{1}} \right)+A_{2}\exp\left( -\frac{t}{\tau_{2}} \right)+A_{0}$ (A14)

where:

$A_{1}=\frac{5k_{PH1}^{2}-5k_{PH1}k_{PH2}-5k_{12}k_{PH1}P_{1}+k_{12}k_{t}P_{1}P_{2}}{5k_{PH1}^{2}-5k_{PH1}k_{PH2}}$ (A15)$A_{2}=\frac{5k_{12}k_{PH2}P_{1}-k_{12}k_{t}P_{1}P_{2}}{5k_{PH1}k_{PH2}-5k_{PH2}^{2}}$ (A16)$A_{0}=\frac{k_{12}k_{t}P_{1}P_{2}}{5k_{PH1}k_{PH2}}$ (A17)

and experimental lifetimes are the same as model ones:

$\tau_{1}=\frac{1}{k_{PH1}}$, (A18)$\tau_{2}=\frac{1}{k_{PH2}}$. (A19)

Out of the three equations A15-A17, only two are linearly independent since the third one may be calculated from the normalization condition: A_1_ + A_2_ + A_0_ = 1. These amplitudes are identical to normalized experimental amplitudes.

Eq. A13 allow more precise correction for extinction coefficients than formulas used previously (Gibasiewicz et al. 2013a, 2013b).

Thus, four linearly independent equations A5, A6, A15, A16 interrelate seven model-based parameters, $k_{12},k_{t},k_{dir1},k_{dir2},k_{\mathrm{PB}},\Delta G_{1},\Delta G_{2}$ (Fig. 2), with five parameters obtained from the two-exponential fitting, τ_1_, τ_2_, *A*_0_-*A*_2_ (Table 1)_._

In particular, from eqs. A15-A17, the following useful formulae for the k_12_P_1_ and k_t_P_2_ products may be derived:

$k_{12}P_{1}=k_{PH1}- A_{1}k_{PH1}- A_{2}k_{PH2}$ (A20)
$k_{t}P_{2}=\frac{5\left( 1-A_{1}-A_{2} \right)k_{PH1}k_{PH2}}{\left( \left( 1-A_{1} \right)k_{PH1}+ A_{2}k_{PH2} \right)}$ (A21)

Finally, it is important to notice that the temperature-dependent lifetimes, τ_T1_ and τ_T2_, in Fig. 2 may be calculated from the formulae (compare to formulae A5 and A6):

$k_{T1}=k_{\mathrm{PB}}\left( 1-P_{1} \right)$, (A22)

$k_{T2}=k_{\mathrm{PB}}\left( 1-P_{2} \right)$. (A23)

**Table S1.** Model parameters for charge recombination kinetics.

| Sample | T [K] | **Model parameters**^(1)^ | | | | | | | |
| --- | --- | --- | --- | --- | --- | --- | --- | --- | --- |
|  |  | Op-tion | **τ_12_ [ns]** | τ_t_ [ns] | ΔG_1_ [meV] | ΔG_2_ [meV] | τ_dir1_ [ns] | τ_dir2_ [ns] | τ_PB_ [ns] |
| WT | 298 | *a* | **4.3±1.1** | 29±10 | *90±18* | *128±26* | 23±55 | 110±430 | *0.2* |
|  |  | *a'* | **3.7±1.0** | 29±10 | *44±9* | *85±17* | 23±36 | 120±390 | *1.0* |
|  |  | *b* | **4.2±1.1** | 29±10 | 77±17 | 119±14 | *150±30* | | *0.26±0.15* |
|  | 78 | *a* | **6.1±1.5** | 35±12 | *90±18* | *128±26* | 10±2 | 103±75 | *0.2* |
|  |  | *a'* | **6.1±1.5** | 35±12 | *44±9* | *85±17* | 10±2 | 103±75 | *1.0* |
|  |  | *b* | **6.0±1.5** | 35±12 | 25±4 | 48±16 | *150±30* | | *0.26±0.15* |
| ELL | 296 | *a* | **6.5±2.1** | 46±15 | *0* | *250* | 35±10 | | 0.54±0.10 |
|  |  | *b* | **9.1±3.7** | 46±15 | 22±14 | 131±14 | *100±20* | | *0.32±0.10* |
|  | 78 | *a* | **5.0±1.6** | 30±10 | *0* | *250* | 62±33 | | 0.68±0.13 |
|  |  | *b* | **7.7±2.8** | 30±10 | 7.9±3.3 | 42±10 | *100±20* | | *0.32±0.10* |
| YLH | 296 | *a* | **1.9±0.5** | 56±19 | *0* | *250* | 21±5 | | 1.30±0.29 |
|  |  | *b* | **3.7±1.0** | 56±19 | 79±16 | 140±16 | *100±20* | | *0.11±0.07* |
|  | 78 | *a* | **1.5±0.4** | 30±10 | *0* | *250* | 65±36 | | 0.97±0.22 |
|  |  | *b* | **2.9±0.8** | 30±10 | 19±4 | 50±11 | *100±20* | | *0.11±0.07* |

^(1)^Model parameters were estimated from the fit parameters of the nanosecond kinetics shown in Table 1 and the formulae shown in the Appendix. Assumed values are shown in italics (in most cases, ranges of assumed values were considered). Uncertainties of the model parameters were estimated using the formula: $u\left( y \right)=\sqrt{\sum_{i} \left( u\left( x_{i} \right) \frac{\partial y}{\partial x_{i}} \right)^{2}}$, where $y$ is any of the model parameters, $x_{i}$ is $i$-th independent variable (fit parameter), $u\left( x_{i} \right)$ is uncertainty of $i$-th independent variable (assumed to be ±20%). Prior to uncertainty estimations, formulas for model parameters were transformed as a function of independent variables obtained directly from the fit, which were $A_{1},A_{2},A_{0},\tau_{1},\tau_{2}$.

**Fig. S1.** Comparison of two possible energetic diagrams describing charge recombination in the ELL RC. Diagrams (a)and (b) correspond to the two sets of model parameters estimated from the nanosecond experiment and presented for the ELL RC in Table 2 (options *a* and *b*, respectively; see also Tab. S1 for the remaining parameters). (a) The free energy gaps between (P^+^H_A_^-^)_1_ or (P^+^H_A_^-^)_2_ and P^+^B_A_^-^ were assumed to be temperature-independent and fixed at 0 and 250 meV, respectively. (b) The free energy gaps between (P^+^H_A_^-^)_1_ or (P^+^H_A_^-^)_2_ and of P^+^B_A_^-^ were free parameters, and it was assumed that τ_PB_ = 0.32 ns and τ_dir_ = ~100 ns. In all cases τ_dir_ = τ_dir1_ = τ_dir2_. Assumed lifetimes are shown in italics.
